# Augmentation or substitution: defining role of large language model in physical education

**DOI:** 10.3389/fspor.2025.1662056

**Published:** 2025-11-12

**Authors:** Huijun Yang, Bingyi Huang, Xu Li

**Affiliations:** School of Physical Education, Collaborative Innovation Team for the Study and Interpretation of Xi Jinping's Important Discourses on Cyberspace Governance, Chongqing University of Posts and Telecommunications, Chongqing, China

**Keywords:** large language model, physical education, artificial intelligence, assistive tools, sustainability teaching

## Abstract

**Introduction:**

Large language model has seen rapid uptake in education alongside advances in artificial intelligence. Its capabilities in areas such as healthcare planning and question answering suggest strong potential for supporting personalised instruction in physical education. At the same time, studies have raised concerns regarding safety, accuracy, and contextual appropriateness. This study examines the role of large language model in physical education and evaluates its suitability as an assistive tool or possible alternative to human instruction.

**Methods:**

This study conducted a questionnaire—based evaluation comparing three generative approaches for producing physical education lesson plans and teacher responses: (1) plans written directly by a large language model, (2) plans produced by a large language model after being provided with domain resources, and (3) plans created collaboratively by physical education teachers together with the large language model. Feasibility, practicality, safety, adaptability, and content quality were assessed across these approaches. Large language model—generated responses in typical physical education scenarios were further evaluated in terms of response accuracy, safety, clarity, adaptability, guidance quality, acceptability to learners, and perceived potential to enhance teacher response efficiency.

**Results:**

Lesson plans produced collaboratively by physical education teachers and the large language model outperformed those produced solely by the large language model—both with and without additional resources—across all evaluated dimensions. In addition, responses generated by the large language model in physical education scenarios were rated highly for clarity, guidance, adaptability, and support for teacher efficiency.

**Discussion:**

The large language model demonstrates clear value in physical education, particularly as a means to support and augment instructional design and responsiveness. However, it does not yet replace the pedagogical judgment, contextual awareness, and safety oversight of human instructors. Overall, this study concludes that the large language model is best positioned as an assistive tool to optimise teaching practice rather than as a standalone replacement for human teachers.

## 1 Introduction

Physical education programmes differ from knowledge-based programmes in the way they teach and promote the development of students' physical and motor skills, primarily through specific physical activities (1). This type of teaching must be based on scientific program design, professional sports instruction, and timely responses to students' questions. The role of physical education teachers therefore extends to multiple dimensions: they are not only instructors in the classroom but also designers of exercise programmes and answerers of questions (2).

With the wide application of artificial intelligence in education, physical education is gradually ushering in a new trend of personalised learning (3). Personalised physical education emphasises the design of teaching activities based on each student's physical fitness, skill level, and learning needs (4). However, physical education faces many challenges, especially the limited resources of teachers. Many teachers, when faced with large classes, often find it difficult to develop individualised exercise plans for each student and lack sufficient time and energy to answer students' specific questions individually during the teaching process (5). This situation may lead to students not being able to get timely answers to the questions they have when performing physical exercise, thus affecting their motivation and effectiveness (6).

Large language models (e.g., ChatGPT, DouBao, Deepseek) have the potential to solve the problems mentioned above as an innovative solution (7). Large language models have demonstrated excellent capabilities in the field of natural language processing by learning from a large amount of literature and data, allowing them to provide cross-domain knowledge and solve specialized problems (8). Meanwhile, large language models have also demonstrated excellent task planning and execution capabilities through advanced algorithms and model architectures (9).

Although, large language models have shown strong potential and application value in several research areas such as natural language (10), biology (11, 12), chemistry (13, 14), etc. Similarly, they also show a broad application potential in the field of education investigated (15–17). However, the impact of large language models in the field of physical education has not been fully. This is despite the fact that previous studies have assessed the ability of large language models to simplify administrative tasks and improve the quality of question responses (18). However, we still need to further explore and validate the potential of large language models to assist or replace physical education teachers in developing lesson plans and answering student queries.

To assess the potential of large language model in physical education, this study compares the differences between three different generation methods: direct generation by ChatGPT 5 Thinking, ChatGPT 5 Thinking generation after feeding the appropriate material, and collaborative generation by the teacher and large language model. This study also investigated ChatGPT 5 Thinking's performance in answering students' questions about physical education learning. The goal of this comparison and investigation is to determine whether ChatGPT 5 Thinking is effective in assisting or replacing physical education teachers in developing lesson plans and responding to students' questions.

## 2 Literature review

### 2.1 Large language models and teacher roles

Education widely uses large language models due to their powerful natural language processing capabilities, extensive coverage of knowledge bases, and automated generation and analysis of textual content. Large language models have significantly contributed to innovation and change in the education industry (19), as well as having a profound impact on the role of teachers (20).

Many studies have used large language model as a teacher's assistant to help teachers with automated, tedious, and time-consuming tasks in order to reduce workloads and increase teaching effectiveness (21). It plays a crucial role in addressing high teacher stress and burnout (22). For instance, teachers can reduce the assessment burden by using large language model to correct students' essays or answer questions, providing detailed feedback and grading (23). Large language model can also automatically generate exam questions, reducing the task of teachers to manually construct questions (24). In addition, large language model provides real-time feedback on students' learning and responses, customising study plans and practice questions for each student to help them master knowledge more effectively (25). Teachers can also use large language model to set learning objectives, generate teaching materials, and recommend appropriate learning resources (26). These features significantly enhance the efficiency and personalisation of the education process, providing strong support for smart education.

There are also some research attempts to explore the potential of large language models for replacing traditional teacher functions. For example, large language models are used as virtual teachers to support intelligent tutoring systems for online teaching through deep learning techniques (27). It explains students' queries and provides personalised responses. This application significantly enhances the educational experience. In addition, students can use large language models for informal English learning by engaging in two-way verbal and textual interactions with dialogue agents via mobile devices (28). This style of learning is not only flexible and convenient, but it also provides continuous learning support and real-time feedback to help students master the target language more effectively (29). Large language models, as a virtual teacher instead of a traditional teacher, can provide great flexibility in terms of time and space and significantly improve the efficiency and effectiveness of learning through personalised instruction. These innovative applications demonstrate the broad prospects of large language models in education, with great potential to drive the digitalization and personalisation of education.

In addition, there are studies that focus on teacher-large language model collaboration, suggesting that the combination of teachers and large language models can create a more effective learning experience than either working alone (30).

Research on the impact of large language models in education has yielded some results. However, research on clearly defining their role as a supplement or substitute in physical education remains lacking. In addition, how physical education teachers use large language models and the nature of the relationship between large language models and physical education teachers have not been adequately investigated. Therefore, this study compares the effectiveness of teachers working collaboratively with large language models and the effectiveness of large language models working alone in terms of developing lesson plans to clarify the potential for the practical use of large language models and their roles in physical education teaching and learning. Through this comparative study, we hope to provide a clearer understanding of the application of large language model in physical education and provide guidance for future educational practice.

### 2.2 Response function of large language models

Large language models can answer a wide range of complex questions due to their powerful natural language processing capabilities and interactive features. Currently, a large number of studies have evaluated the performance of large language models in answering questions in the health and medical domains (31–33). While sports research has received less attention, the medical field's findings hold equal relevance. Research has shown that large language models have significant potential to improve information access and decision support. For example, Sezgin et al. (34) compared two publicly accessible large language models (GPT-4 and LaMDA) with the Google search engine. The results showed that the GPT-4 had generally higher quality responses, outperforming the other models, especially in terms of clinical accuracy, while there was no significant difference in response quality between LaMDA and Google Search. Breneman et al. (35) analysed the robustness of ChatGPT responses to cirrhosis and hepatocellular carcinoma treatments and associated emotional support, concluding that ChatGPT can be used as an adjunctive information tool for both patients and physicians to improve clinical outcomes. Chen et al. (18)'s study showed that responses to large language models can reduce physician workload, increase the consistency of physicians' responses, and enhance the informational and educational value of responses.

However, there are also studies that point to a degree of risk associated with responses from large language models (36). Breneman et al. (35) found that while large language models may provide some useful pre-operative information, there are also irrelevant details and inaccuracies. Chen et al. (18) warns that large language models may accidentally alter clinical decision-making, and that doctors may rely on the assessment of large language models rather than making independent judgements, which can affect the accuracy of medical decisions. Therefore, Chen et al. (18) advises physicians and patients to use large language models' responses and resources with caution. Accordingly, the present study referred to previous studies and included an investigation of the performance of large language models in responding to questions about sport, with a view to exploring the value and limitations of their potential application in this field.

## 3 Methods

### 3.1 Research design

This study began with a comparative analysis approach in which lesson plans for a single physical education lesson were generated using three different modalities to assess the role of the large language model and its role in the development of lesson plans for physical education courses. The first way was to directly use ChatGPT 5 Thinking to generate a complete physical education course lesson plan, through which we wanted to understand the performance of the basic teaching programme that large language model can provide without any external information input. The second way is more complex, after inputting a detailed course syllabus, a course profile, a description of the teaching venue and equipment, as well as the textbook University Physical Education Curriculum and a number of reference books including Exercise Training, Exercise Physiology, Exercise Nutrition, Exercise Psychology, Exercise Injury Prevention and Rehabilitation, Exercise Prescribing, and Exercise Testing and Evaluation, and then generating the teaching plan using ChatGPT 5 Thinking lesson plans. This approach was designed to assess the performance of large language models' generated lesson plans when supported by specific background information and resources. Experienced physical education teachers, working in collaboration with large language models, modified and improved the lesson plans generated in the second approach, forming the basis of the third approach. We used this as a comparison to evaluate the outcomes of large language model and human experts working together.

To ensure the comparability of the three different generation methods, we developed a standardised lesson plan template before the start of the experiment. The template encompassed modules that addressed the target audience, lecture topics, teaching objectives, teaching focus, teaching difficulties, teaching methods, teaching tools, course structure, teaching content, organisation and pedagogy, timetable, assignments, venue equipment, and safety plans. The task of the large language model in the development of the lesson plan is mainly to develop and adjust the content of the lesson plan without involving changes in the structure of the template.

Additionally, we designed a series of question-and-answer dialogues on physical activity to further explore large language model performance in response to student query responses. These Q&A dialogues focused on sports injury issues and covered five main categories: open soft tissue injuries, closed soft tissue injuries, joint and ligament injuries, fractures, and sports fatigue. Through these dialogues, we hoped to assess the potential of the large language model in dealing with sports injury-related questions and whether it could effectively assist teachers in answering students' related questions.

### 3.2 Participants

The participants in this study were 12 experienced physical education teachers, all of whom have been teaching physical education for more than 10 years and all of whom hold the title of professor. We selected participants based on their senior status in the field of physical education and their deep understanding of curriculum design, teaching methods, and student needs, enabling this study to gain a more comprehensive and in-depth insight into a diverse educational environment.

These faculty members came from seven different colleges and universities and possessed a wide range of teaching backgrounds and expertise. By selecting these experienced teachers as research subjects, this study aims to ensure the reliability and wide applicability of the findings in order to more objectively assess the complementary or alternative roles of large language model in physical education teaching and to analyse the effectiveness of its application and potential problems in actual teaching scenarios, so as to provide a scientific basis and a practical guide for the future application of large language model in the field of education.

This study was approved by the Ethics Committee Review Board of Chongqing University of Posts and Telecommunications (Approval No. 202406130102696). All participants provided written informed consent prior to participation.

### 3.3 Data collection

This study collected the ratings of 12 physical education teachers using a questionnaire. For lesson plan development comparison, the questionnaire was based on a 5-point Likert scale covering satisfaction ratings from 1 to 5, with 1 being “very dissatisfied” and 5 being “very satisfied.” The questionnaire collected expert ratings of feasibility, practicality, safety, adaptability, and quality of contents, each of which contained multi-dimensional questions, to comprehensively assess the differences in the performance of the lesson plans generated in the three different ways. To avoid subjective bias in expert ratings caused by differences in generation methods, we hid the names of the three generation methods and replaced them with numbers. Large language model directly generates Method 1, large language model generates Method 2 after feeding the corresponding materials, and teachers collaborate with large language model to generate Method 3. For the responses to the students' questions, this study employed an 11-point semantic differential scale. The questionnaire collected ratings in seven areas, including accuracy, safety, clarity, adaptability, guidance, acceptability, and efficiency improvement.

### 3.4 Data analysis

This study employed various data analysis methods to ensure the reliability and validity of the results. Prior to formal data analysis, the collected questionnaire results underwent reliability and validity testing. The results indicated that the Cronbach's Alpha coefficient was 0.947 and the Kaiser-Meyer-Olkin value was 0.715 (*p* < 0.05), suggesting that the data's reliability and validity were sound.

For the comparison of plans, the Shapiro–Wilk test results for each variable indicated that the data were normally distributed, thus a one-way ANOVA was employed. Additionally, the Levene test results showed that the variances between sample data were homogeneous (*P* > 0.05). However, for the analysis of each dimension within the variables, since the group data were ordinal and did not conform to a normal distribution (*P* < 0.05), we used non-parametric analysis methods, specifically the Kruskal–Wallis test. When the Kruskal–Wallis H test indicated that at least one group was significantly different from the others (*p* < 0.05), we further applied Dunn's Multiple Comparisons Test for pairwise comparison analysis to precisely identify which categories had significant differences.

For the analysis of responses to student questions, descriptive statistical methods were employed, and the results were visualized. Through these methods, we could intuitively present the responses to student questions, providing data support for further analysis and discussion.

## 4 Results

### 4.1 Lesson plan

The ANOVA analysis results (see Table 1) demonstrate significant inter-group differences among the three generation methods in terms of feasibility (*F* = 55.939), practicality (*F* = 87.836), safety (*F* = 128.003), adaptability (*F* = 38.468), and content quality (*F* = 94.891) (*p* < 0.05). Additionally, the mean square values for the within-groups were relatively small, indicating minimal within-group variability and suggesting a high level of agreement among the expert ratings.

**Table 1:** ANOVA results of lesson plans.

Measure	Source	Sum of squares	Mean square	*F*	Sig.
Adaptability	Between Groups	880.056	440.028	55.939	.000
	Within Groups	259.583	7.866		
	Total	1,139.639			
Feasibility	Between Groups	684.056	342.028	87.836	.000
	Within Groups	128.500	3.894		
	Total	812.556			
Safety	Between Groups	1,428.722	714.361	128.003	.000
	Within Groups	184.167	5.581		
	Total	1,612.889			
Practicality	Between Groups	234.889	117.444	38.468	.000
	Within Groups	100.750	3.053		
	Total	335.639			
Contents quality	Between Groups	377.167	188.583	94.891	.000
	Within Groups	65.583	1.987		
	Total	442.750			

The results of Tukey HSD multiple comparisons analyses showed (see Table 2) that all pairwise comparisons were significant (*p* < 0.05), further validating that there were significant differences among the three generation methods. When Method 3 as (I) Group was compared pairwise with methods 1and 2, the values of Mean Difference (I–J) were all positive, indicating that Method 3 was the best performer on all assessment indicators. In contrast, when Method 1 as (I) Group was compared pairwise with Method 2 and Method 3, the values of Mean Difference (I–J) were all negative, indicating that Method 1 performed the worst on all assessment metrics.

**Table 2:** Tukey HSD multiple comparison results for lesson plans.

Dependent variable	(I) Group	(J) Group	Mean difference (I–J)	Std. error	Sig.	95% confidence interval	
						Lower bound	Upper bound
Adaptability	1	2	−3.333*	1.145	.017	−6.14	−.52
		3	−11.750*	1.145	.000	−14.56	−8.94
	2	1	3.333*	1.145	.017	.52	6.14
		3	−8.417*	1.145	.000	−11.23	−5.61
	3	1	11.750*	1.145	.000	8.94	14.56
		2	8.417*	1.145	.000	5.61	11.23
Feasibility	1	2	−4.917*	.806	.000	−6.89	−2.94
		3	−10.667*	.806	.000	−12.64	−8.69
	2	1	4.917*	.806	.000	2.94	6.89
		3	−5.750*	.806	.000	−7.73	−3.77
	3	1	10.667*	.806	.000	8.69	12.64
		2	5.750*	.806	.000	3.77	7.73
Safety	1	2	−5.583*	.964	.000	−7.95	−3.22
		3	−15.250*	.964	.000	−17.62	−12.88
	2	1	5.583*	.964	.000	3.22	7.95
		3	−9.667*	.964	.000	−12.03	−7.30
	3	1	15.250*	.964	.000	12.88	17.62
		2	9.667*	.964	.000	7.30	12.03
Practicality	1	2	−4.000*	.713	.000	−5.75	−2.25
		3	−6.167*	.713	.000	−7.92	−4.42
	2	1	4.000*	.713	.000	2.25	5.75
		3	−2.167*	.713	.013	−3.92	−.42
	3	1	6.167*	.713	.000	4.42	7.92
		2	2.167*	.713	.013	.42	3.92
Contents quality	1	2	−4.333*	.576	.000	−5.75	−2.92
		3	−7.917*	.576	.000	−9.33	−6.50
	2	1	4.333*	.576	.000	2.92	5.75
		3	−3.583*	.576	.000	−5.00	−2.17
	3	1	7.917*	.576	.000	6.50	9.33
		2	3.583*	.576	.000	2.17	5.00

* The mean difference is significant at the 0.05 level.

#### 4.1.1 Feasibility

Table 3 presents the results of the Kruskal–Wallis tests showed that the three generation methods were significantly different (*p* < 0.05) on all four dimensions of feasibility. Further Dunn's multiple comparisons test showed that method 3 was significantly better than method 1 in terms of frequency, intensity, duration, and reasonableness of type of exercise (F1), but not significantly different from method 2. In terms of ease of access to resources (F2), ease of understanding and implementation (F3), and consideration of practical conditions (F4), method 3 performed the best, followed by method 2, while method 1 performed the worst on these dimensions. These results further validate the significant advantages of method 3 in practical applications.

**Table 3:** Comparative results of feasibility.

Items	Methods	Mean rank	Kruskal–Wallis		Methods		
			H	Asymp. sig.	1	2	3
F1	1	9.58	17.464*	.000	1	0.088227361	8.83335 × 10^−05^
	2	18.75			0.088227361	1	0.136577919
	3	27.17			8.83335 × 10^−05^	0.136577919	1
F2	1	7.63	28.816*	.000	1	0.042119653	2.47197 × 10^−07^
	2	17.88			0.042119653	1	0.010998116
	3	30			2.47197 × 10^−07^	0.010998116	1
F3	1	8.92	22.173*	.000	1	0.096345837	7.70476 × 10^−06^
	2	17.92			0.096345837	1	0.031432202
	3	28.67			7.70476 × 10^−06^	0.031432202	1
F4	1	9.75	21.603*	.000	1	0.285009714	1.32126 × 10^−05^
	2	16.75			0.285009714	1	0.010441843
	3	29			1.32126 × 10^−05^	0.010441843	1

* The mean difference is significant at the 0.05 level.

#### 4.1.2 Practicality

Further analysing the differences on the four dimensions of practicality, the results of the Kruskal–Wallis tests in Table 4 show that no significant differences were found between the three generation methods (*H* = 0.22, *p* > 0.05) in terms of “improving physical fitness and health” (P1), suggesting that the practicality of these generation methods is similar on this dimension. The remaining dimensions of the Kruskal–Wallis tests were significantly different (*p* < 0.05). The Dunn's Multiple Comparisons Test revealed that method 1 significantly outperformed methods 2 and 3, while there was no significant difference in scores between methods 2 and 3. Method 3 was significantly better than method 1, but not significantly different from method 2 in terms of “focusing on long-term exercise habits and healthy lifestyle development of students” (P3).

**Table 4:** Comparative results of practicality.

Items	Methods	Mean rank	Kruskal–Wallis		Methods		
			H	Asymp. sig.	1	2	3
P1	1	17.5	0.22	.896	/	/	/
	2	19.38			/	/	/
	3	18.63			/	/	/
P2	1	6.83	24.763*	.000	1	0.001237584	4.71627 × 10^−06^
	2	21.67			0.001237584	1	0.61235025
	3	27			4.71627 × 10^−06^	0.61235025	1
P3	1	14.88	7.284*	.026	1	1	0.043261313
	2	15.83			1	1	0.081278363
	3	24.79			0.043261313	0.081278363	1
P4	1	8	20.874*	.000	1	0.006126839	2.44345 × 10^−05^
	2	20.88			0.006126839	1	0.505233842
	3	26.63			2.44345 × 10^−05^	0.505233842	1

* The mean difference is significant at the 0.05 level.

#### 4.1.3 Safety

The results of the Kruskal–Wallis tests (see Table 5) showed that the three generation methods differed significantly on all seven dimensions of safety (*p* < 0.05). The results of Dunn's Multiple Comparisons Test showed that Method 1 was significantly worse than Method 2 and Method 3 in terms of the scores of “safety practices and emergency plans” (S1), while Method 2 and Method 3 did not differ significantly. In “Emergency Planning” (S1), method 1 performed significantly worse than methods 2 and 3, whereas there was no significant difference in the scores of methods 2 and 3. Method 3 was significantly better than Method 1 for Physical Health Assessment Requirements (S2) and Safety Standards for Sites and Equipment (S5), but it was not significantly different from Method 2. Method 3 was significantly better than Method 1, but not significantly different from Method 2, in terms of “mental health assessment requirements” (S3), “students' personal medical history, existing injuries, or limitations” (S4), “adequate rest and recovery schedules” (S6), “high risk of injury or illness” (S7), and “high-risk manoeuvres or activities” (S7). Method 3 was significantly better than methods 1 and 2, but there was no significant difference in the ratings between methods 1 and 2.

**Table 5:** Comparative results for safety.

Items	Group	Mean rank	Kruskal–Wallis		Methods		
			H	Asymp. sig.	1	2	3
S1	1	6.92	23.451*	.000	1	0.000277298	2.83231 × 10^−05^
	2	23.21			0.000277298	1	1
	3	25.38			2.83231 × 10^−05^	1	1
S2	1	11.46	13.577*	.001	1	0.347671563	0.000721832
	2	17.79			0.347671563	1	0.107241127
	3	26.25			0.000721832	0.107241127	1
S3	1	10.67	25.34*	.000	1	1	6.72015 × 10^−06^
	2	14.33			1	1	0.000345877
	3	30.5			6.72015 × 10^−06^	0.000345877	1
S4	1	10.33	24.862*	.000	1	0.785617399	5.57085 × 10^−06^
	2	15			0.785617399	1	0.000797747
	3	30.17			5.57085 × 10^−06^	0.000797747	1
S5	1	12.08	11.483*	.003	1	0.519959101	0.002269793
	2	17.63			0.519959101	1	0.134380263
	3	25.79			0.002269793	0.134380263	1
S6	1	9.46	21.368*	.000	1	0.151787615	1.23692 × 10^−05^
	2	17.54			0.151787615	1	0.024134095
	3	28.5			1.23692 × 10^−05^	0.024134095	1
S7	1	10.21	27.82*	.000	1	0.768094811	1.48231 × 10^−06^
	2	14.79			0.768094811	1	0.000297322
	3	30.5			1.48231 × 10^−06^	0.000297322	1

* The mean difference is significant at the 0.05 level.

#### 4.1.4 Adaptability

Further analysing the differences in the eight dimensions of adaptability (see Table 6), the Kruskal–Wallis tests found no significant differences between the three generation methods in adapting to the “age and gender characteristics of the students” (*H* = 1.551, *p* > 0.05), suggesting that these generation methods are similar in adapting to the students' basic demographic characteristics. For the other seven dimensions, the results of the Kruskal–Wallis tests showed significant differences (*p* < 0.05). The results of Dunn's Multiple Comparisons Test showed significant differences (*p* < 0.05) between the “Personal Fitness Level” (A2) and the “Instructional Environment”. In “Teaching and learning environment” (A5), method 3 was significantly better than method 2, but there was no significant difference between the scores of method 1 and methods 2 and 3. Method 1 was significantly worse than Methods 2 and 3 in terms of “personal health status” (A3), while there was no significant difference between Methods 2 and 3. In terms of 'support measures for students with special needs' (A4), Method 3 was significantly better than Methods 1 and 2, while there was no significant difference between Methods 1 and 2. Method 3 was significantly better than Method 1 but not significantly different from Method 2 in terms of “catering for diverse interests and needs” (A6) and “progressive improvement” (A7). Method 3 was the best performer, and method 2 was the second-best performer in terms of “adaptation and optimization based on real-time feedback” (A8), while method 1 was the worst performer in these dimensions.

**Table 6:** Comparison results for adaptability.

Items	Methods	Mean rank	Kruskal–Wallis		Methods		
			H	Asymp. sig.	1	2	3
A1	1	19.04	1.551	.46	/	/	/
	2	15.75			/	/	/
	3	20.71			/	/	/
A2	1	19.17	12.143*	.002	1	0.143432739	0.405228117
	2	11			0.143432739	1	0.001541575
	3	25.33			0.405228117	0.001541575	1
A3	1	10.96	10.158*	.006	1	0.030837296	0.010503645
	2	21.54			0.030837296	1	1
	3	23			0.010503645	1	1
A4	1	9.33	23.839*	.000	1	0.209876405	4.04542 × 10^−06^
	2	16.83			0.209876405	1	0.007575844
	3	29.33			4.04542 × 10^−06^	0.007575844	1
A5	1	17.75	7.813*	.02	1	0.79192702	0.290459663
	2	13.13			0.79192702	1	0.01643295
	3	24.63			0.290459663	0.01643295	1
A6	1	12.17	7.932*	.019	1	0.168428427	0.018019139
	2	19.96			0.168428427	1	1
	3	23.38			0.018019139	1	1
A7	1	10.17	16.518*	.000	1	0.173638903	0.000146386
	2	18.13			0.173638903	1	0.09122846
	3	27.21			0.000146386	0.09122846	1
A8	1	7.83	26.46*	.000	1	0.030337222	8.07178 × 10^−07^
	2	18.5			0.030337222	1	0.030337222
	3	29.17			8.07178 × 10^−07^	0.030337222	1

* The mean difference is significant at the 0.05 level.

#### 4.1.5 Contents quality

The results of the Kruskal–Wallis tests in Table 7 show that the three generation methods differed significantly (*p* < 0.05) on all four dimensions of content quality. The results of Dunn's Multiple Comparisons Test revealed that method 1 significantly underperformed method 1 in terms of being “based on the latest exercise science and health theories” (CQ1) and “with appropriate depth to challenge students' fitness and skills” (CQ3). “up-to-date exercise science and health theories” (CQ1) and “has appropriate depth to challenge students' fitness and skills” (CQ3), method 1 was significantly worse than methods 2 and 3, while there were no significant differences between methods 2 and 3. In terms of “covering a wide range of motor skills and health knowledge” (CQ2), method 3 was significantly better than method 1, but not significantly different from method 2. In terms of “providing different levels of challenge and support” (CQ4), method 3 was significantly better than methods 1and 2, while there was no significant difference between methods 1and 2.

**Table 7:** Comparative results of contents quality.

Items	Methods	Mean rank	Kruskal–Wallis		Methods		
			H	Asymp. sig.	1	2	3
CQ1	1	7.75	22.637*	.000	1	0.007237373	8.16037 × 10^−06^
	2	20.42			0.007237373	1	0.292689322
	3	27.33			8.16037 × 10^−06^	0.292689322	1
CQ2	1	12.54	9.222*	.01	1	0.56392234	0.007381175
	2	17.96			0.56392234	1	0.260925238
	3	25			0.007381175	0.260925238	1
CQ3	1	7.29	22.806*	.000	1	0.001280947	1.58173 × 10^−05^
	2	21.96			0.001280947	1	0.907868691
	3	26.25			1.58173 × 10^−05^	0.907868691	1
CQ4	1	11.25	19.405*	.000	1	0.953655072	7.49357 × 10^−05^
	2	15.42			0.953655072	1	0.003897285
	3	28.83			7.49357 × 10^−05^	0.003897285	1

* The mean difference is significant at the 0.05 level.

### 4.2 Responses to questions

**Figure 1:**
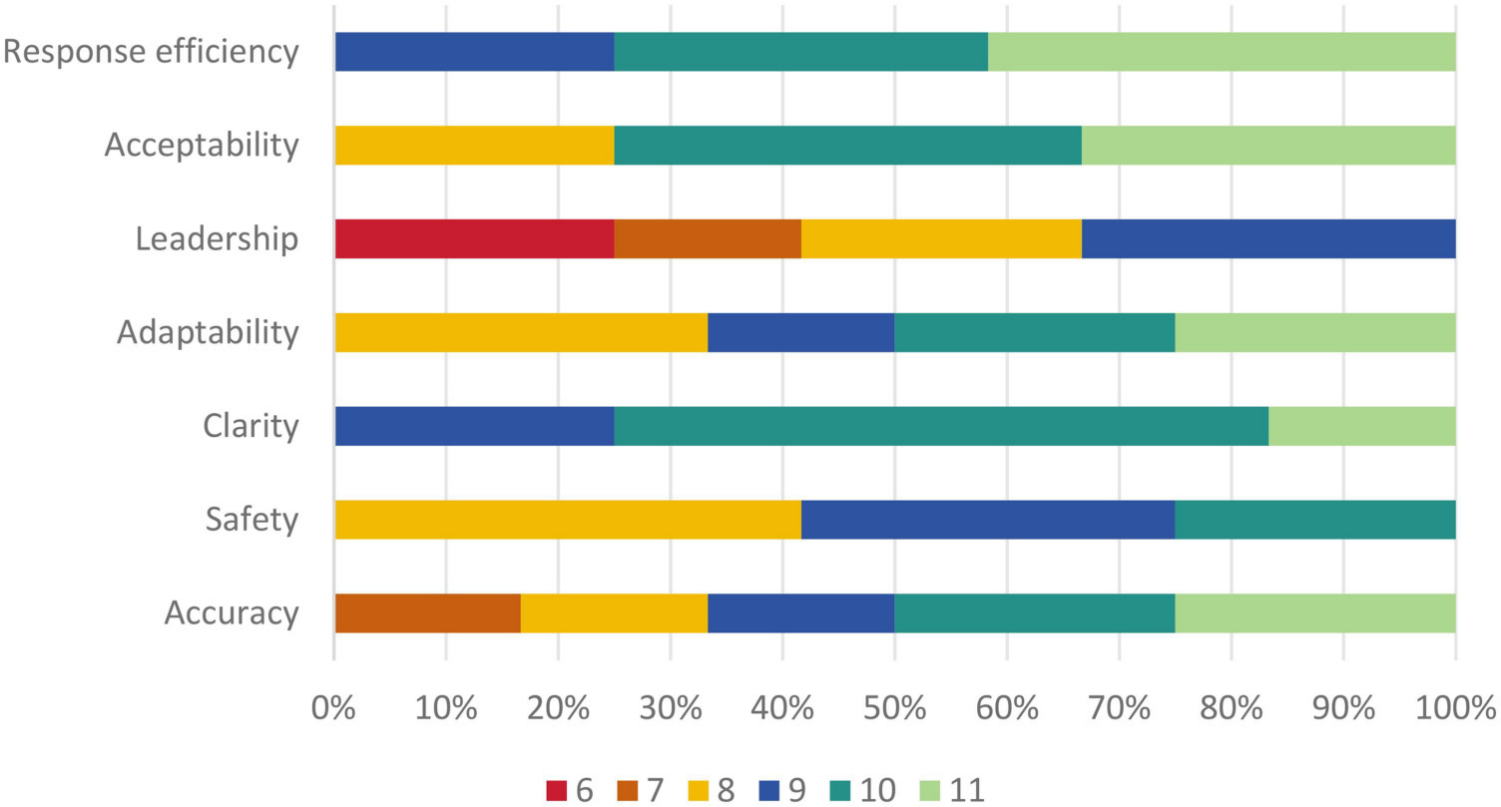
Distribution of ratings for question responses.

The results of the descriptive statistics (see Table 8) show that the experts rated the overall performance of the “dialogue pairs of question responses” as high, indicating that the large language model performs well as a support tool for physical education teachers. However, the results also revealed shortcomings in motivating students to participate in physical activities and handling certain details of information. Figure 1 shows the percentage ratings for the seven assessment indicators. There were no ratings below 6 in any of the results. The warm-coloured areas in the figure represent areas with medium ratings, whereas the cool-coloured areas indicate areas with high ratings. This visualisation provides an intuitive view of how the large language model performs on each of the assessment metrics.

**Table 8:** Descriptive statistics results of question responses.

Descriptive statistics	Accuracy	Safety	Clarity	Adaptability	Leadership	Acceptability	Response efficiency
Mean	9.25	8.83	9.92	9.42	7.67	9.83	10.17
Median	9.50	9.00	10.00	9.50	8.00	10.00	10.00
Std. Deviation	1.485	.835	.669	1.240	1.231	1.193	.835
Minimum	7	8	9	8	6	8	9
Maximum	11	10	11	11	9	11	11
Sum	111	106	119	113	92	118	122

In the survey on the “correctness and reliability of the sport-related information and answers provided by large language model,” most experts (50 percent) gave a high rating of 10 or higher, indicating that large language model was able to provide high-quality responses. However, a further 16.7 percent of experts felt that large language model's performance was only worth a score of 7, noting that there were deficiencies in certain details. This result suggests that although large language model performs well in the provision of information, there is still room for improvement, especially in the detailing of information.

Most ratings on the survey “Does the response provided by large language model take into account the safety of students in sport to avoid potential risk of injury” were centred on scores of 8 and above (75%), indicating that large language model has a good track record of considering the safety aspects of sport. However, it's important to note that no experts awarded full scores, implying that despite some acknowledgment, sports safety instruction still requires improvement.

In the survey on “large language model's clarity, comprehensibility, and expressiveness in communicating with students, especially in explaining exercise techniques and precautions,” 25.0 percent of the experts gave a rating of 9, 58.3 percent of the experts gave a rating of 10, and 16.7 percent of the experts gave a rating of 11. The positive ratings from all experts who participated in the survey indicate that large language models are generally considered to be clear, understandable, and highly articulate when communicating with students, especially when explaining sports skills and precautions.

In the survey on “large language model's ability to adapt to students' different needs and levels,” 33.3 percent of experts gave a rating of 8, 16.7 percent gave a rating of 9, 25.0 percent gave a rating of 10, and 25.0 percent gave a rating of 11. Overall ratings were more dispersed. Only 58.3 percent of the experts gave a rating of 10 or above, indicating that although large language model performs well in terms of adaptability, some experts still believe that there is room for improvement.

In the survey on “the effectiveness of large language model in guiding and motivating students to participate in sports activities,” more than a third of the experts thought large language model performed well in this area, giving it a high rating of 9 points. However, 41.7 percent of experts still considered it to be underperforming, giving it a low rating (6 or 7). No expert gave a perfect rating of 10 or 11. This indicates that there is still room for improvement in the large language model's ability to guide students in responding to their queries as an assistant to the physical education teacher.

In the survey on “the degree of acceptance and recognition of the answers provided by large language model,” 41.7 percent of the experts gave a high rating of 11, indicating that they considered large language model to be almost impeccable in terms of the efficiency of its responses and that it was able to provide answers in a prompt and efficient manner. The fact that 33.3 percent of the experts gave a score of 10 shows that they are very satisfied with the speed and efficiency of large language model's responses and believe that large language model is able to provide the required information in a timely manner, while 25 percent of the experts gave a score of 9, which may imply that they believe that large language model's responses, although very efficient, may have room for improvement in some cases.

In the survey on “whether large language model improves the efficiency of physical education teachers' responses and reduces their burden,” 41.7 percent of the experts gave a high rating of 11, 33.3 percent gave a rating of 10, and 25 percent gave a rating of 9. The experts generally gave very positive ratings, indicating that experts generally believe that large language model is of great value in enhancing the efficiency of physical education teachers and reducing their workload.

## 5 Discussion

### 5.1 Lesson plans designed by large language model

By comparing three different generation methods of physical education lesson plans and investigating the performance of large language models' responses to the questions, this study aims to understand the potential for practical application and the role of large language model in physical education. The study showed that lesson plans generated by physical education teachers in collaboration with large language models performed best in terms of feasibility, practicality, safety, adaptability, and content quality. This result is consistent with the findings of the Mishra et al. (37) study and highlights the importance of optimising prompts and incorporating expert judgement when using large language models. In addition, we noted that in some respects (e.g., F1, P1, P2, P3, P4, S1, S2, S5, A1, A3, A6, A7), there was no significant difference between “collaborative generation by physical education teachers and large language model” and “large language model generation after feeding the appropriate resources.” There was no significant difference between them. This result is consistent with the findings of Boiko et al. (38), who noted that AI systems powered by GPT-4 are capable of semi-autonomously designing, planning, and executing science experiments in multiple steps. This suggests that large language model has the potential to replace the work of physical education teachers on some specific tasks. However, these substitution roles are limited to specific areas of work and do not completely replace the full range of duties and roles of physical education teachers (19). Therefore, we can infer that the large language model has the potential to serve as an auxiliary tool or assistant, effectively supporting physical education teachers in the development of teaching or training plans.

Large language models not only reduce physical education teachers' workload, but they also optimize the effectiveness of teaching and training by providing high-quality advice and scenarios, making educational practice more precise and efficient. Singh et al. (39) research has demonstrated that adding natural language annotations to explain upcoming actions can improve task success rates for generating planning procedures. This approach allows for subsequent iterative updates of the plan, enhancing the flexibility and adaptability of the system. Similarly, Yang et al. (40) demonstrated an large language model-based framework that enables robots to continuously reason and update plans based on the latest environmental changes, outperforming previous work in responding to various environmental changes and accomplishing diverse tasks (41). Teachers can achieve personalised planning based on individual student profiles by leveraging the powerful computational and processing capabilities of large language models, significantly improving the feasibility of this burdensome task. This collaborative work model not only reflects the broad application potential of AI in physical education, but it also provides strong support for future in-depth cooperation between physical education teachers and intelligent systems.

### 5.2 Large language model responses to questions and answers

Meanwhile, the results of large language model's response survey indicated that large language model excelled at answering questions related to sports learning, particularly in terms of response efficiency, clarity, and acceptability. Experts generally agreed that large language model's responses to questions posed by students were to the point, informative, and comprehensive, with clear and understandable language, demonstrating its excellent qualities as an AI assistant. This result is consistent with the findings of previous studies. For example, Chen et al. (18) concluded that large language models improved the accuracy and consistency of responses while reducing teacher workload and enhancing the informative and educational value of responses. Kieser et al. (42) noted that ChatGPT 5 Thinking excelled in problem-solving accuracy, with responses approaching those of human teachers. Deng et al. (36) further emphasised that the use of large language models not only increased teachers' efficiency in answering students' questions but also enabled teachers to devote more time and energy to other important aspects of teaching and student guidance, resulting in an overall improvement in teaching quality and teachers' job satisfaction. As a result, it is reasonable to infer that large language model can serve as an effective tool or assistant to help physical education teachers answer students' questions. By introducing large language model, physical education teachers are not only able to provide accurate and detailed answers to students more efficiently, but they also significantly reduce the time it takes for students to obtain the information they need. This technological assistance not only enhances teaching effectiveness and efficiency, but also improves students' learning experience and sports participation.

In addition, we should be wary of the limitations that exist with large language model. The study found that the results generated directly using large language model were worse in all aspects than the results generated by physical education teachers in collaboration with large language model. Moreover, neither the plans generated by physical education teachers in collaboration with large language model nor the plans generated by large language model after feeding the appropriate resources received a perfect score in terms of safety. The experts observed that there is still significant room for improvement in the practical application of the current large language model, limiting its use to a supplementary tool rather than a complete replacement for physical education teachers' work. This finding is consistent with previous research. Deng et al. (36) stated that despite the promising future of large language models, an optimistic and cautious approach is crucial for the safe integration of large language models into the application environment. They called for a rigorous evaluation of large language models, noting that large language models face challenges including disillusionment, a lack of transparency, and consistency. Killian et al. (43) said that ignoring or disregarding predictions about the capabilities of technologies such as AI catboats could lead us to make “stupid decisions.” Pavlik (44) emphasized that to address these risks, it is important to clearly recognize the limitations of large language model and use it only as a tool to support and enhance learning, not as a substitute for human authority and other sources of authority. These analyses lead us to recommend a more cautious approach in the use of large language model in physical education. Physical education teachers should avoid over-relying on large language model-generated results and employ critical thinking to use large language model as a supportive tool rather than as the primary basis for decision-making.

## 6 Conclusion

This study examined the role of the large language model in the context of physical education training and assessed its potential and limitations as a supplementary or alternative tool. The study demonstrates that huge language models largely serve as a supplementary tool in physical education instruction, rather than completely replacing human teachers. Firstly, the study found that large language model shows substitution potential in some respects, but its best application is as a supplementary tool, in collaboration with physical education teachers, to promote the development of physical education teaching and learning. Being optimistic but cautious ensures that we capitalise on the benefits of the technology while avoiding potential risks and challenges. Therefore, we advocate the application of large language model in physical education teaching and learning to assist physical education teachers in tasks such as lesson plan development and answering difficult questions.

We recommend providing large language model with relevant materials or links to resource libraries, or setting some restrictions, to further enhance its effectiveness in physical education. This measure will enable large language model to support physical education teachers more effectively, thus optimising all aspects of physical education. Large language model can improve the efficiency and quality of teaching and learning by integrating a wealth of resources, while also allowing physical education teachers to focus on more valuable teaching interactions and personalized instruction. This move not only makes full use of the technological advantages of large language model but also maximises teaching and learning outcomes. As a result, we advocate for improving large language models' adjunctive functions by systematically providing comprehensive and precise resource support in physical education, thereby contributing to the overall advancement of the field.

Despite the significant assistive potential demonstrated by large language models in physical education teaching, the vision of a complete replacement of human teachers is not realistic. It is crucial to exercise caution when evaluating large language model-generated content to prevent over-reliance on its output. While large language models can provide a wealth of knowledge support and personalised advice, they cannot replace the unique role that human teachers have in key areas such as practical guidance, emotional communication, and motivation. In addition, it is important to be wary of the potential for large language model to produce erroneous or confusing information to prevent it from adversely affecting physical education instruction. As a result, we advocate for maintaining critical thinking when using large language model, ensuring that it is used as an aid, not a substitute, to optimise the overall effectiveness of physical education instruction.

Future research should focus on exploring and developing more effective integration strategies that utilise large language models as powerful adjunct tools. This includes in-depth research on how to better combine the strengths of large language models and human teachers in physical education to achieve complementary advantages and thus enhance the overall teaching effect. By making rational use of the knowledge support and personalised advice from large language models, physical education teachers can focus more on interaction and personalised instruction in actual teaching, thus significantly improving the quality and effectiveness of teaching. Future research should focus on building a collaborative working framework to ensure close cooperation between large language models and human teachers, so that technology and human intelligence can complement each other and jointly promote the progress and development of physical education.

## Data Availability

The raw data supporting the conclusions of this article will be made available by the authors, without undue reservation.
