# Supplementary material for: Augmentation or substitution: defining role of large language model in physical education
Source: Front Sports Act Living. 2025 Nov 12;7:1662056. doi: 10.3389/fspor.2025.1662056 (PMC12647000; doi:10.3389/fspor.2025.1662056)
Supplement: Supplementary file 1 [file Datasheet1.docx]

Category: Adaptability

1. Does the plan align with learners’ age and sex characteristics?

2. Can it be adjusted to learners’ individual fitness levels?

3. Can it be adjusted to learners’ individual health status?

4. Does it include accommodations for learners with special needs?

5. Is it adaptable to different instructional environments?

6. Does it offer multiple exercise modalities and activities to meet diverse interests and needs?

7. Does it provide graded difficulty levels to support progressive improvement in fitness?

8. Can it be adjusted and optimized based on real-time feedback?

Category: Feasibility

1. Are the frequency, intensity, time, and type (FITT) parameters reasonable?

2. Are required resources (e.g., equipment and facilities) readily available?

3. Are there clear, step-by-step procedures and operational guidelines that are easy to understand and implement?

4. Does it account for the institution’s practical constraints (e.g., budget and facilities)?

Category: Safety

1. Does it include detailed safety procedures and emergency plans?

2. Are there recommendations or requirements for physical health screening?

3. Are there recommendations or requirements for mental health screening?

4. Does it consider learners’ medical history, existing injuries, or limitations?

5. Does it consider safety standards for venues and equipment?

6. Does it include adequate rest and recovery scheduling?

7. Does it include any high-risk movements or activities?

Category: Practicality

1. Does it improve learners’ fitness and health?

2. Does it incorporate objective assessments (e.g., fitness tests, body-composition measures) to track individual progress?

3. Does it emphasize the development of long-term exercise habits and healthy lifestyles?

4. Is there a mechanism for periodic evaluation and improvement of the instructional plan?

Category: Content Quality

1. Is it scientifically accurate and grounded in current exercise science and health theory?

2. Is it comprehensive, covering multiple movement skills and health knowledge areas?

3. Does it have appropriate depth to challenge learners’ fitness and skills?

4. Is it appropriate for different proficiency levels, providing sufficient challenge and support?
