# Supplementary material for: Augmentation or substitution: defining role of large language model in physical education
Source: Front Sports Act Living. 2025 Nov 12;7:1662056. doi: 10.3389/fspor.2025.1662056 (PMC12647000; doi:10.3389/fspor.2025.1662056)
Supplement: Supplementary file 2 [file Datasheet2.docx]

# ****LLM Prompts and Settings (for Reproducibility)****

## B.1 Model identifiers and access

1. **Provider / model:** ChatGPT 5 Thinking (OpenAI).
2. **Access method:** ChatGPT web interface (no plug-ins; no external tools).
3. **Version/build:** 2025.06 (stable channel).
4. **Model access dates:** 2025.09-2025.10
5. **Context window:** 128,000 tokens.
6. **Generation language:** English.

No third-party agent framework was used. Outputs were generated directly via the ChatGPT interface.

## B.2 Fixed inference parameters

| **Parameter** | **Plan Generation (Modalities 1–2)** | **Q&A Responses** | **Rationale** |
| --- | --- | --- | --- |
| Temperature | **0.30** | **0.20** | Lower variance for safety/consistency in Q&A |
| Top-p | **0.90** | **0.90** | Standard nucleus sampling |
| Max tokens (per response) | **2,000** | **1,200** | To avoid overlong outputs |
| Frequency penalty | **0.00** | **0.00** | Default |
| Presence penalty | **0.00** | **0.00** | Default |
| Stop sequences | **None** | **None** | — |
| Seed | **Unset (provider default)** | **Unset** | — |
| Safety filters | **Provider defaults: ON** | **Provider defaults: ON** | Disallowed content enforced |

## B.3 Global system instruction (verbatim; all modalities)

**System message**
You are a certified physical education instructor and exercise-science specialist. Produce evidence-aligned guidance that prioritizes participant safety, clarity, feasibility, and adaptability in real-world instructional settings. Follow these rules: (1) state assumptions explicitly; (2) when information is insufficient, indicate limits or request the minimum clarifying detail; (3) include contraindications, red-flag symptoms, and referral guidance where risk is non-trivial; (4) align exercise selection and FITT parameters with declared goals, constraints, and available equipment; (5) avoid clinical diagnosis and defer medical decisions to licensed clinicians; (6) present outputs in a concise, structured, instructional format (headings, bullets, progression criteria).

## B.4 Representative prompts by modality

### B.4.1 Modality 1 — ****General LLM (no domain inputs)****

**User prompt (plan generation).**
Using the template below, generate a 60-minute physical education session for **first-year university students (mixed sex)** whose goals are **foundational movement, aerobic base, and injury prevention**. Available equipment: **cones, mats, resistance bands**. Venue: **indoor court**. Provide: session goals; warm-up; main sets with movements, sets×reps or time, and rest; coaching cues; regression/progression options; safety and contraindications; cooldown; and brief evaluation. Keep the exact template structure and do not add new sections.

**Template provided to the model (fixed across conditions).**

1. Target population and goals
2. Session structure (timeline)
3. Exercise selection and progression
4. Intensity/volume/load prescription (FITT)
5. Coaching cues (key points; common faults)
6. Safety & contraindications; emergency plan
7. Equipment and venue notes
8. Adaptations for constraints (time, space, ability)
9. Session evaluation criteria

**User prompt (Q&A).**
Answer as a PE coach. Prioritize safety and evidence-aligned guidance. If uncertain, state limits and suggest referral. Student profile: **19-year-old male, novice**. Question: “**I rolled my ankle (mild swelling) yesterday. Can I do lower-body training today?**” Provide: short answer; rationale; do/don’t list; modifications; when to stop and seek care.

### B.4.2 Modality 2 — ****LLM with domain resources (curated context supplied)****

**Context block prepended to conversation (excerpt).**

1. **Course syllabus:** University Physical Education (semester outline; weekly objectives; assessment overview).
2. **Course profile & constraints:** class size ≈ 30; 60-minute sessions; indoor court; mixed fitness levels.
3. **Venue/equipment:** court dimensions; available cones, mats, resistance bands; no barbells.
4. **Reference list (titles/years):** University Physical Education Curriculum (latest ed.); Exercise Training; Exercise Physiology; Exercise Nutrition; Exercise Psychology; Exercise Injury Prevention and Rehabilitation; Exercise Prescription; Exercise Testing and Evaluation.
5. **Use policy:** When guidance conflicts, prioritize safety and mainstream consensus from the listed sources; state assumptions and uncertainty.

**User prompt (plan generation).**
Using the supplied syllabus, venue/equipment, and references, generate a 60-minute session for **first-year university students** with goals **foundational movement and injury prevention**. Follow the standard template. Ensure FITT parameters are feasible for **class size 30** and include progressions/regressions and safety checks relevant to **indoor court** and **band-based resistance**.

**User prompt (Q&A).**
Using the supplied references and constraints, answer the student’s question: “**My knees hurt when I squat; how should I adjust depth and stance?**” Provide: brief answer; rationale; two regressions; one progression; red-flags requiring cessation/referral.

### B.4.3 Modality 3 — ****LLM + expert collaboration (expert-guided revision)****

**User prompt (expert revision—plan).**
Here is a draft session generated with the same template. Review it as a senior PE instructor. Improve specificity of exercise selection and progressions, tighten safety guidance (contraindications, red-flags, referral), and ensure feasibility for **class size 30** with **bands/mats**. Keep the template structure unchanged. Provide a bullet-point change log explaining each revision and its rationale (safety, feasibility, clarity, adaptability).

**User prompt (expert revision—Q&A).**
Here is a draft answer to the student’s question. Strengthen safety caveats, clarify coaching language, and add two context-appropriate regressions and one progression. Conclude with a brief “when to stop/seek care” checklist.

**Blinding note:** Raters received anonymized artifacts labeled **Method 1/2/3**. Prompts, context blocks, and modality identifiers were not visible during rating.

## B.5 Safety and scope guardrails (applied in all modalities)

**Provider safety filters:** ON (default).

**Scope limits embedded in system instruction:** no diagnosis or treatment; defer medical decisions; include red-flags and referral triggers (e.g., suspected fracture, severe pain, neurological symptoms, syncope).

**Uncertainty handling:** when evidence is limited or profile details are missing, the model states assumptions and proposes conservative, lower-risk options.

## B.6 Reproducibility artifacts packaged with this appendix

1. **Verbatim system and user prompts** for each modality (plan; Q&A).
2. **Parameter record** for each generation (temperature, top-p, max tokens, context window, penalties).
3. **Scenario bank** (exact student profiles and question texts; see Methods Table 2).
4. **Standardized lesson-plan template** (see Methods).
